# Patient Perspectives in Cancer Surgery

**DOI:** 10.3390/jcm11030789

**Published:** 2022-01-31

**Authors:** David Martin, Nicolas Demartines, Martin Hübner

**Affiliations:** Department of Visceral Surgery, University Hospital CHUV, University of Lausanne (UNIL), 1011 Lausanne, Switzerland; demartines@chuv.ch (N.D.); martin.hubner@chuv.ch (M.H.)

Cancer patients frequently misunderstand essential information, incorrectly state the extent of their disease, are unclear about the treatment goal, and overestimate their prognosis [1,2,3,4]. Similarly, care providers are largely unaware of their patients’ needs and priorities, which are rarely discussed in depth in clinical routine. Finding a common language and mutual understanding of treatment goals is important in order to define the optimal treatment for the individual patient, whatever the medical or surgical specialty [5]. For patients with advanced disease, these conversations should also touch on taboos such as treatment intent, prognosis, and end of life discussions [4,6]. However, it is difficult to define how much and what kind of information is required to fulfil the wishes or needs of patients, to allow them to make a truly informed decision and to bring shared decision making to the next level. Patients and care providers can have quite different views on aims and results of a treatment due to large interindividual and cultural differences. Furthermore, physicians’ information on expected benefits but also potential risks and side effects can have a large influence on the patients’ decision [7].

Historically, surgical quality and effectiveness were assessed by clinical outcome measures, like morbidity, mortality, and readmission rates [8]. In cancer surgery, additional metrics like resection margins, quality of lymphadenectomy, or overall and disease-free survivals are among the other common comparative measures described in the literature and used for decision-making in daily routine. Patient-reported outcome measures (PROMs) go beyond these traditional clinical and oncological measures parameters. PROMs ascertain perceptions of the patient’s health status, perceived impairment, disability, and quality of life. The integration of this seemingly obvious but in fact novel concept starts to induce a shift in how the healthcare system and associated stakeholders think of quality in cancer surgery [9]. Patients should also have the opportunity to define their goals and expectations for the care that they want to receive; however, this aspect seems to be often missing in current clinical practice. Adopting a true shared decision-making process would arguably allow the tailoring of treatment strategies, avoid unnecessary treatments, and optimize the use of limited resources. This could be as beneficial and important to patients, families, and care providers as the expensive development of new molecules and treatments. The quality of care could also be assessed through the patients’ experience of the treatment. Patient-reported experience measures (PREMs) gather information on patient’s views of their care or a health service, using satisfaction scales in particular, whereas PROMs provide reports from patients about their own health [10]. Patients may report a good outcome but a bad experience (and vice versa). The same complication can induce very different feelings and reactions in patients depending on the circumstances, communication, and presence of the care provider team.

The integration of PROMs and PREMs in outcome assessment has improved patient satisfaction, enhanced communication among patients and their care teams, and helps to improve quality of life and survival [9,11]. Challenges for routine implementation of these important measures include costs, resources, and logistical issues. Once these problems are overcome, the question of the type of PROM and PREMs arises: General? Cancer-specific? Site-specific? In which language? Then, the method of delivery can play a role: Paper? Electronic? Computer/Tablet/Smartphone? And finally, how often should they be re-assessed? 

PROMs and PREMs are important without any doubt, but their translation in everyday clinical practice requires further investigations and development of standardized methodology. There is also an increasing role of technology to support the delivery, completion, and scoring of surveys, which may lead to more efficient clinical intervention [9]. Furthermore, patient acceptance and participation, as well as physician and support staff acceptance, should be measured in future prospective and qualitative studies. As William J. Mayo already mentioned in 1910: “The best interest of the patient is the only interest to be considered and in order that the sick may have the benefit of advancing knowledge, union of forces is necessary”.

The present Special Issue on “Patient Perspectives in Cancer Surgery” aims to explore patient-reported experience and outcomes measurements in cancer surgery in order to improve the global care of patients.
